# Macroinvertebrate diversity and ecosystem functioning across the eutrophication gradients of the middle and lower reaches of Yangtze River lakes (China)

**DOI:** 10.1002/ece3.9751

**Published:** 2023-01-18

**Authors:** Yongjiu Cai, Rui Dong, Giri Kattel, You Zhang, Kai Peng, Zhijun Gong

**Affiliations:** ^1^ Key Laboratory of Watershed Geographic Sciences Nanjing Institute of Geography and Limnology, Chinese Academy of Sciences Nanjing China; ^2^ University of Chinese Academy of Sciences Beijing China; ^3^ School of Geographical Sciences Nanjing University of Information Science and Technology Nanjing China; ^4^ Department of Infrastructure Engineering The University of Melbourne Melbourne Victoria Australia; ^5^ Department of Hydraulic Engineering Tshinghua University Beijing China

**Keywords:** biodiversity, biomass, ecosystem function, eutrophication, macroinvertebrates diversity, shallow lakes

## Abstract

Biodiversity, which strengthens ecosystem stability, ecosystem function, and ecosystem services, is threatened by anthropogenic perturbation and climate change worldwide. However, despite the study of the role of biodiversity in multiple facets of freshwater ecosystems, the linkages between macroinvertebrates diversity and ecosystem functioning have not yet been well‐assessed in eutrophication gradients of lowland river‐floodplain systems. In this study, we have examined the relationship between macroinvertebrates diversity (species diversity, functional diversity, phylogenetic diversity) and macroinvertebrates biomass across the three typically featured eutrophication gradients: “*MACROPHYTE,*” “*TRANSITION,*” and “*PHYTOPLANKTON,*” of floodplain lakes in the middle and lower reaches of the Yangtze River (China). Our results suggest that macroinvertebrates diversity in three different lacustrine conditions, biomass, and the relationship between diversity and biomass varied along eutrophication gradients. Functional richness and variance (divergence in taxon community) were the two important macroinvertebrate diversity indices, which accounted for the largest amount of variation in the biomass (63% in *PHYTOPLANKTON* lakes and 57% in *MACROPHYTE* lakes, respectively). We also found that the macrophyte coverage is more important than the relative abundance in maintaining the macroinvertebrates diversity and biomass in lowland Yangtze floodplain lake systems, while the relative abundance of macrophyte would change the BEF relationship. Our results demonstrate the functional performance of Yangtze River lakes, which would change with increased nutrient loading and decreased macrophyte coverage and would highlight the significance of the restoration of macrophytes to reduce nutrient loads.

## Graphical abstract

The relationship between macroinvertebrates diversity (species diversity, functional diversity, phylogenetic diversity) and macroinvertebrates biomass varied across eutrophication gradients. Macrophyte cover presence is important in maintaining macroinvertebrates diversity and biomass, while the relative abundance of macrophyte would change the biodiversity and ecosystem function (BEF) relationship. This study highlights the indispensable role of macrophytes in macroinvertebrates diversity, the dynamics of biomass, and the BEF relationship in Yangtze River lakes.

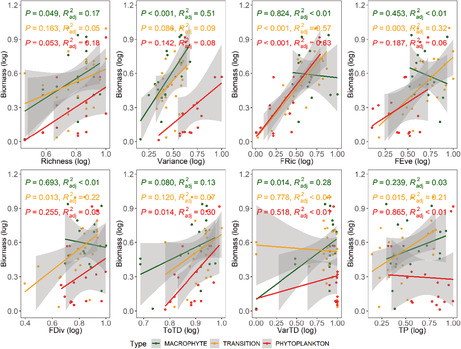

## 1 INTRODUCTION

The aquatic biodiversity is rapidly changing due to eutrophication, with potentially large and even irreversible consequences in the ecosystem structure and functioning worldwide (Dalu et al., 2022; Kattel, 2022; Qin et al., 2013; Reid et al., 2019; Zhang et al., 2021). Especially the freshwater ecosystems have become the most vulnerable and threatened in regions where humans have persistently intensified their use for various purposes (Reid et al., 2019). The links between biodiversity and ecosystem functioning have been explored for more than two decades, but studies exploring the relationships between biodiversity and ecosystem function (BEF) across trophic gradients in freshwater ecosystems are limited (Covich et al., 2004; Dahlin et al., 2021).

Macroinvertebrates diversity plays an important role in maintaining ecosystem functioning, such as resistance to disturbances, biomass production, and leaf decomposition in riverine and lacustrine systems (Manning et al., 2018). As consumers at intermediate trophic levels, macroinvertebrates regulate nutrient cycling, primary productivity, secondary productivity, decomposition, and translocation of materials (Covich et al., 2004). Actions such as sediment burrowing and mixing by macroinvertebrates can transform organic detritus from sedimentary storage into dissolved nutrients as a major source of primary producers (Covich et al., 2004; Dahlin et al., 2021). Indices such as species richness, Shannon diversity, and Simpson diversity of macroinvertebrates all are proven to be positive indicators of the organic matter breakdown and nutrient cycling in lakes and rivers (Cao et al., 2018; Smeti et al., 2019). Functional diversity of macroinvertebrates has been found to be even more robust than taxonomy‐based index to explain the strength of ecosystem functioning in lacustrine and riverine systems (Cadotte et al., 2011). Phylogenetic diversity (PD) is especially a powerful dimension to explain the variation in the ecosystem functioning as this effectively unravels the biological trait measurement (Le Bagousse‐Pinguet et al., 2019). Evidence shows that functional diversity and phylogenetic diversity together better explain the variation in the ecosystem functioning. For instance, trait diversification and phylogenetic diversity have greater niche complementarity in macroinvertebrates reflecting the enhanced functional specialization in freshwater systems (Cadotte et al., 2011; Le Bagousse‐Pinguet et al., 2019). Despite extensive studies on macroinvertebrates diversity and its effects on ecosystem functioning, only a few, such as the litter decomposition and nutrient cycling are limited in lakes and rivers (Dahlin et al., 2021; Monroy et al., 2017).

The macroinvertebrates communities in floodplain lake ecosystems maintain multiple functions simultaneously, which are often mediated by various drivers. While biodiversity strongly influences ecosystem functioning (Cao et al., 2018; Monroy et al., 2017), environmental factors can also directly and indirectly affect macroinvertebrates diversity and communities in floodplain lakes. Most studies to date, however, have taken advantage of showcasing natural variations across multiple sites to explore the effect of various environmental drivers on macroinvertebrates diversity (Dong et al., 2021; Zhang et al., 2019). Studies also have focused on how environmental drivers cause macroinvertebrates biomass change in floodplain lake systems (Dou et al., 2022; Li, Jiang, et al., 2019; Li, Liu, et al., 2019; Zhang et al., 2019). However, the links between biodiversity and the environmental factors influencing macroinvertebrates biomass under varying nutrient‐enriched conditions of multiple floodplain lakes have not yet been addressed comprehensively. Hence, for understanding how the macroinvertebrates diversity is mediated by nutrient enrichment in floodplain lakes, and consequently how the eutrophication condition would influence macroinvertebrates biomass, needs the use of a multifaceted approach of ecological tests. In such an approach, the links between macroinvertebrates diversity and their biomass across the extended eutrophication gradients of floodplain lakes are needed to explore further.

Many lakes in the lower Yangtze River basin are shallow and have gone through conditions related to eutrophication and other water‐related pollution issues for decades as they are located mostly around the densely urbanized and industrialized areas (Zhang et al., 2019). Yet, there is a low to high degree of nutrient enrichment given the variations in the anthropogenic perturbation in these floodplain lakes over time (Zhang et al., 2019). The clear water, macrophyte‐dominated lakes usually prevail low nutrient levels as higher coverage of macrophyte improves water transparency by reducing sediment resuspension, trapping periphyton and competing for nutrients with algae and providing a refuge for macroinvertebrates and zooplankton against fish predation (Kattel et al., 2018). While there are also lakes in transition, the macrophyte‐dominated lakes are gradually transformed into algal‐dominated lakes due to persistent eutrophication over time. Besides, some lakes have already gone through severe eutrophication with high nutrient enrichment, increased wind‐driven sediment resuspension, and densely bloomed filamentous algae causing shading effects on underwater vegetation, consequently lowering macroinvertebrate and other invertebrate diversity and assemblages (Kattel et al., 2018). Increased human interventions including river regulation for hydropower and irrigation development have also profoundly implicated for ecological dynamics of the middle and lower reaches of Yangtze River lakes further (Kattel et al., 2016).

**FIGURE 1:**
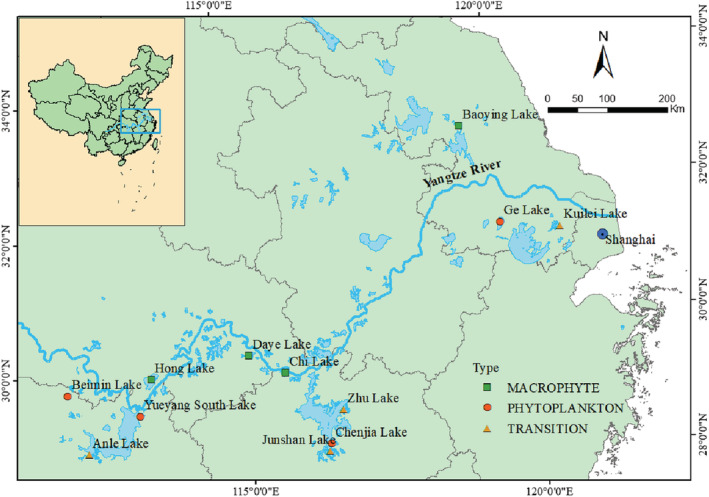
Location of study lakes in the middle and lower basin of the Yangtze River.

Being influenced by a range of environmental drivers (warming, wastewater discharge, land use change and nutrient) and with the increased complexity of ecosystem function and dynamics, understanding the linkages between macroinvertebrate diversity and biomass in lower Yangtze River lakes would provide an important insight into tackling pollution, and management challenges of lake eutrophication in the region. Hence, the structure and dynamics of the community and functional diversity of macroinvertebrates in these lakes would be invaluable evidence to unravel the current state of ecosystem functioning and regulating ecosystem services. Here we have compared community structure, species diversity, functional diversity, and phylogenetic diversity as a measure to evaluate the biomass of benthic macroinvertebrates in three typical shallow lake ecosystems: “*MACROPHYTE*,” “*TRANSITION*,” and “*PHYTOPLANKTON*” along eutrophication gradients in the lower Yangtze River lakes (Figure 1). We hypothesized that (i) community structure, diversity, and biomass would change along the eutrophication gradient; (ii) diversity—biomass relationship may depend on trophic status; and (iii) eutrophication and other factors that vary along the basin geography would, at least partially, drive the spatial variations in the macroinvertebrates biomass through modulation of biodiversity.

## 2 MATERIALS AND METHODS

### 2.1 Study area

The middle and lower reaches of the Yangtze River are a floodplain area that experiences subtropical monsoon with a mean annual rainfall of above 1000 mm. It is a hotspot biodiversity that interacts with one of the highly developed areas with relatively high levels of urban, agricultural development, and population density (Zhang et al., 2019). Many lakes in the region are threatened by eutrophication and turned from the macrophyte‐dominant type toward the algae‐dominant type. Twelve lakes were selected to represent the three typical lake ecosystems: “*MACROPHYTE*” (*n* = 4), “*TRANSITION*” (*n* = 4), and “*PHYTOPLANKTON*” (*n* = 4) along the middle and lower reaches of the Yangtze River (Table S1). *MACROPHYTE* lakes (Lake Honghu, Lake Chi, Lake Daye, and Lake Baoying) were represented as high in submerged macrophytes cover and low in phytoplankton biomass, *TRANSITION* lakes (Lake Anle, Lake Junshan, Lake Zhu, and Lake Kuilei) were having both low coverage of submerged macrophytes and phytoplankton biomass, while the macrophytes distribution in *PHYTOPLANKTON* lakes (Lake Yueyang South, Lake Chenjia, Lake Ge, and Lake Beimin) was opposite from that of *MACROPHYTE* lakes with a high density of algae (Figure S1; Hu et al., 2016). Three to six sites were sampled in 2012 during summer. Max water depth of each lake was ranging from 1.9 to 7.5 m with mean water depth ranging from 1.1 to 5.5 m (Table S1).

### 2.2 Field sampling

Sampling of macroinvertebrates was conducted in the pelagic zone with 1/16 m^2^ modified Peterson grab, with six grabs comprising a sample. All macroinvertebrates collected from the sampling site were pooled and rinsed in the field to remove fine sediments, and all sediment samples were fixed with 7% buffered formaldehyde solution. In the laboratory, samples were sorted manually by hand on a white enamel pan with the aid of a dissection microscope. Samples were then preserved in 70% ethanol for future use. The macroinvertebrates were identified to the lowest feasible taxonomic level under a dissection microscope (Olympus® SZX10) or a microscope (Olympus® BX53) using regionally available keys for China (Zhang et al., 2019).

Water temperature (WT), pH, and dissolved oxygen were measured in situ, under‐surface water (0.5 m below the water surface) using water quality analyzer (YSI 6600 V2). Secchi depth (SD) was also measured in the field. For each sampling site, the water sample was collected from 0.5 m below the water surface, then kept at 4°C, at the refrigerator laboratory for further chemical analysis. The area of aquatic vegetation (Macrophyte coverage) was estimated and divided into five classes 0%, 0%–5%, 5%–25%, 26%–50%, 51%–75%, and 76%–100%, in the field assigning the classes from 0 to 5 (Zhang et al., 2019).

### 2.3 Laboratory analyses

The water sample was vacuum‐filtered onto a precombusted, weighed glass microfiber filter (Whatman GF/F, Diameter 47 mm) to measure chlorophyll a (Chl‐a). Chlorophyll a, total nitrogen (TN), and total phosphorus (TP) were measured based on standard methods (APHA, 2012) in the Institutional Center for Shared Technologies and Facilities of Nanjing Institute of Geography and Limnology Chinese Academy of Science (NIGLAS).

### 2.4 Diversity and ecosystem function measurement

#### 2.4.1 Species diversity

Species diversity was measured by three indices including species richness (richness), Pielou evenness (evenness), and variance, which represent three multiple facets (i.e., richness, evenness, and divergence) of the community (Magurran, 2004). The indices were computed in *diversity* function using the R package “vegan” (Oksanen et al., 2022).

#### 2.4.2 Functional diversity

A total of 60 functional categories of 10 traits (i.e., maximal size, life cycle duration, aquatic stages, reproduction, dissemination, resistance form, respiration, locomotion and substrate relation, food, feeding habitat) were considered (Table S2). In this study, all 60 functional categories were calculated based on fuzzy‐coded, and affinities for each trait category were standardized as percentage affinities within a trait (Mondy & Usseglio‐Polatera, 2014). We multiplied the taxa trait matrix by the taxa‐site abundance matrix to obtain a traits‐site matrix that included the relative abundance of each trait category in each site (Sarremejane et al., 2017). Functional diversity was measured by three complementary components including functional richness (FRic), functional evenness (FEev), and functional divergence (FDiv). The indices were computed in *dbFD* function using the R package “FD” (Laliberté et al., 2022).

#### 2.4.3 Phylogenetic diversity

Taxonomic distinctness based on the taxonomic relatedness of species is one of the simplest and most widely used indices of phylogenetic diversity (Bhat & Magurran, 2006; Li, Jiang, et al., 2019; Li, Liu, et al., 2019) was used as one of the diversity measurements.

#### 2.4.4 Taxonomic diversity (D)

Average path length between the two randomly selected individuals in a sample was measured.

#### 2.4.5 Taxonomic distinctness (TD)

Average path length between the two randomly selected individuals that are from different species was measured.

#### 2.4.6 Average taxonomic distinctness (AvTD)

Average path length between the two randomly chosen species in a sample based on presence–absence data was measured.

#### 2.4.7 Total taxonomic distinctness (ToTD)

A combination of species and AvTD was measured.

#### 2.4.8 Variation in taxonomic distinctness (VarTD)

The measure was calculated based on presence–absence data (Clarke & Warwick, 2001; Heino et al., 2007). All indices were then computed in *taxondive* function using the R package “vegan” (Oksanen et al., 2022).

#### 2.4.9 Ecosystem functioning measurements

Standing biomass is one of the most used metrics of ecosystem function (Benkwitt et al., 2020; Eger et al., 2021). Macroinvertebrate biomass, one of the good indicators of ecosystem provision and nutrient cycling, and a critical determinant of the functioning of freshwater ecosystems, was measured (Covich et al., 2004).

### 2.5 Statistical analyses

As macroinvertebrates data were not normally distributed so that log(*x* + 1) transformation was performed prior to the analysis. One‐way analysis of the variance was used to compare the difference among environmental parameters, macroinvertebrates diversity, and biomass of various types of lakes (Cai et al., 2017). *T*‐test was used to compare the difference between macrophyte coverage absence and presence data.

We fitted ordinary least squares (OLS) regressions for the effects of diversity and environmental factors on macroinvertebrates biomass. Next, we fitted the GLMs to investigate the best predictors of biomass from both biotic and abiotic factors. To address multicollinearity, we used VIF < 10 to choose variables. We used the dredge function in the R package “MuMIn” to find the candidate models (Bartoń, 2022). We restricted that the maximum number of independent variables to three to avoid over‐fitting is described in the literature (Burnham & Anderson, 2002) and selected the representative predictors.

In addition, because the linear regression and the GLMs assume that the biodiversity and/or abiotic effects are additive, but not interactive, we further fitted piecewise SEM models to explore how eutrophication mediates the relationship between biodiversity and ecosystem function (Grace et al., 2012). We constructed the piecewise SEM models based on the assumption that nutrient inputs either directly or indirectly, through diversity and/or environmental factors, would impact on ecosystem functions. Hence, diversity and environmental factors were selected based on the GLMs results mentioned earlier. We fitted the component models of the piecewise SEM as GLMs models with gaussian and binomial distribution as coverage absence is a categorical variable (Lefcheck, 2016). We reported the standardized coefficient for each path from each component model. The overall fit of the piecewise SEM was evaluated using the Shipley's test of d‐separation: Fisher's *C* statistic and AIC in the R package “piecewise SEM” (Lefcheck et al., 2020).

## 3 RESULTS

### 3.1 Environmental characterization

**FIGURE 2:**
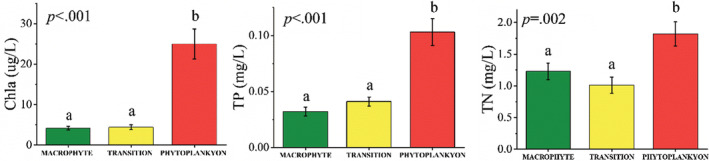
Eutrophication gradients of three types of lakes, *MACROPHYTE*, *TRANSITION*, and *PHYTOPLANKTON* in the middle and lower reaches of the Yangtze River basin.

Significant differences were observed in the SD, pH, Chl‐a, TN, and TP among three lake types (Figure 2, Table S3, *p* < .05). The Chl‐a and TP increased along the eutrophication gradients (Figure S1). The *PHYTOPLANKTON* lakes were measured with the highest Chl‐a (24.97 ± 3.71 μg/L), TN (1.82 ± 0.19 mg/L), and TP (0.103 ± 0.012 mg/L), the lowest SD (0.41 ± 0.05 m) and the absence of macrophyte coverage; while the *MACROPHYTE* lakes were measured with the highest macrophyte coverage (4.17 ± 0.60); and the *TRANSITION* lakes were measured with the highest SD (1.31 ± 0.17 m) and DO (8.6 ± 0.4 mg/L), respectively (Table S3).

### 3.2 Macroinvertebrate community structure, biodiversity, and ecosystem function

One‐way ANOSIM analysis suggests that there were significant differences in the community structure among three types of lakes (*p* < .001). The higher richness of scrapers was found in the *MACROPHYTE* lakes where the dominant taxon groups were Gastropoda and Chironomidae, with the most common species including *Bellamya aeruginosa*, *Parafossarulus striatulus*, *Alocinma longicornis*, *Propsilocerus akamusi*, and *Chironomus flaviplumus*. The *TRANSITION* lakes had the highest collector‐filterer richness, with the dominant species *Corbicula fluminea* among the benthic macroinvertebrate communities. The richness of scrapers and collector‐filterer was lowest in the *PHYTOPLANKTON* lakes exclusively dominated by species of Chironomidae and Oligochaeta such as *Limnodrilus hoffmeisteri, Chironomus flaviplumus*, *Microchironomus tabarui*, respectively (Figure S2; Tables S4–S6).

There were significant differences in the Pielou evenness, variance, FRic, FEve, D, TD, AvTD, macroinvertebrates biomass among the three types of lakes (*p* < .05). The *PHYTOPLANKTON* lakes had the lowest Pielou evenness, FRic, FEve, D, AvTD, ToTD, and the highest variance, VarTD. Significant difference in the ecosystem functioning measurement was observed between the *PHYTOPLANKTON* lakes and the other two types of lakes. For instance, the *PHYTOPLANKTON* lakes had the lowest macroinvertebrates biomass (Table 1). The results attributed high biomass for the species of the Gastropoda group (e.g., *Bellamya aeruginosa, Alocinma longicornis, Parafossarulus striatulus*) and Bivlavia (e.g., *Corbicula fluminea*) in the *MACROPHYTE* and *TRANSITION* lakes, while the macroinvertebrate biomass was less attributed to the *PHYTOPLANKTON* lakes (Table S7).

**TABLE 1:** Macroinvertebrate diversity and ecosystem function in three types of lakes, *MACROPHYTE*, *TRANSITION*, and *PHYTOPLANKTON*, respectively.

Category	Measures	*MACROPHYTE*	*TRANSITION*	*PHYTOPLANKTON*	p
Species diversity	Species richness	5.83 ± 0.44	6.25 ± 0.42	5.94 ± 0.58	.776
	Pielou's evenness	0.82 ± 0.03^a^	0.81 ± 0.02^a^	0.57 ± 0.06^b^	<.001
	Variance	2095.62 ± 916.56^a^	2240.25 ± 493.46^a^	158,621.06 ± 104,358.36^b^	<.001
Functional diversity	FRic	2.53 ± 0.31^a^	2.06 ± 0.23^a^	0.92 ± 0.26^b^	<.001
	FEve	0.53 ± 0.03^a^	0.55 ± 0.04^a^	0.31 ± 0.04^b^	<.001
	FDiv	0.79 ± 0.03	0.71 ± 0.03	0.74 ± 0.03	.261
Phylogenetic diversity	D	48.39 ± 3.59^ab^	57.90 ± 2.35^a^	35.37 ± 5.13^b^	<.001
	TD	69.83 ± 3.95^a^	85.12 ± 2.46^b^	70.28 ± 5.28^ab^	.003
	ToTD	457.23 ± 44.07	508.96 ± 30.85	424.27 ± 45.28	.278
	AvTD	75.40 ± 3.67^ab^	82.95 ± 1.95^a^	72.03 ± 2.54^b^	.002
	VarTD	672.58 ± 82.49^a^	664.79 ± 78.52^a^	1058.18 ± 87.68^b^	.001
Ecosystem functioning	Macroinvertebrates biomass	8.46 ± 1.84^a^	8.49 ± 1.80^a^	4.25 ± 1.79^b^	.007

Different letters indicate signiﬁcant differences among the lake types (*p* < 0.05).

### 3.3 Relationships between biodiversity, abiotic factors, and ecosystem functioning

**FIGURE 3:**
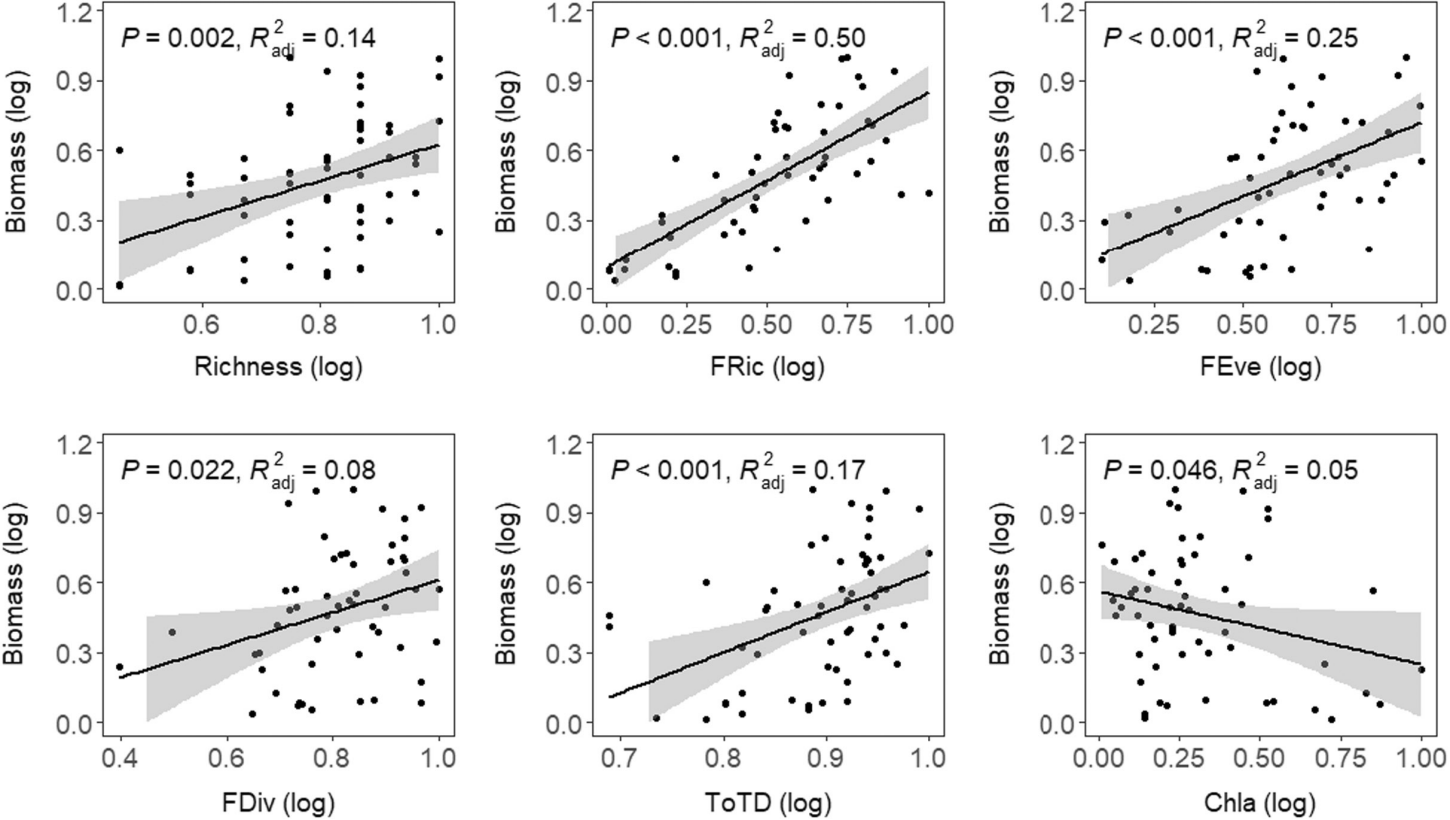
Ordinary least squares (OLS) analysis of the relationship between species diversity, functional diversity, phylogenetic diversity, and ecosystem functioning. The black fitted line is from a linear regression. The results of variance for biodiversity are shown by the *R* ^2^ values. Shaded areas show 95% confidence interval of the fit. Only a significant relationship has been presented.

We fitted OLS regression to explore the influence of abiotic and biotic factors on macroinvertebrates biomass. The richness, FRic, FEve, FDiv, ToTD were found to have a significant positive effect on macroinvertebrates biomass across all sites (*p* < .05), where the adjusted *R* ^2^ were 14%, 50%, 25%, and 8%, respectively. The Chl‐a showed a significant negative effect on macroinvertebrates biomass across all sites (*p* < .05), where the adjusted *R* ^2^ was only 5% (Figure 3). Significant effect of macrophyte coverage absence was found on macroinvertebrates biomass (*p* < .001).

**FIGURE 4:**
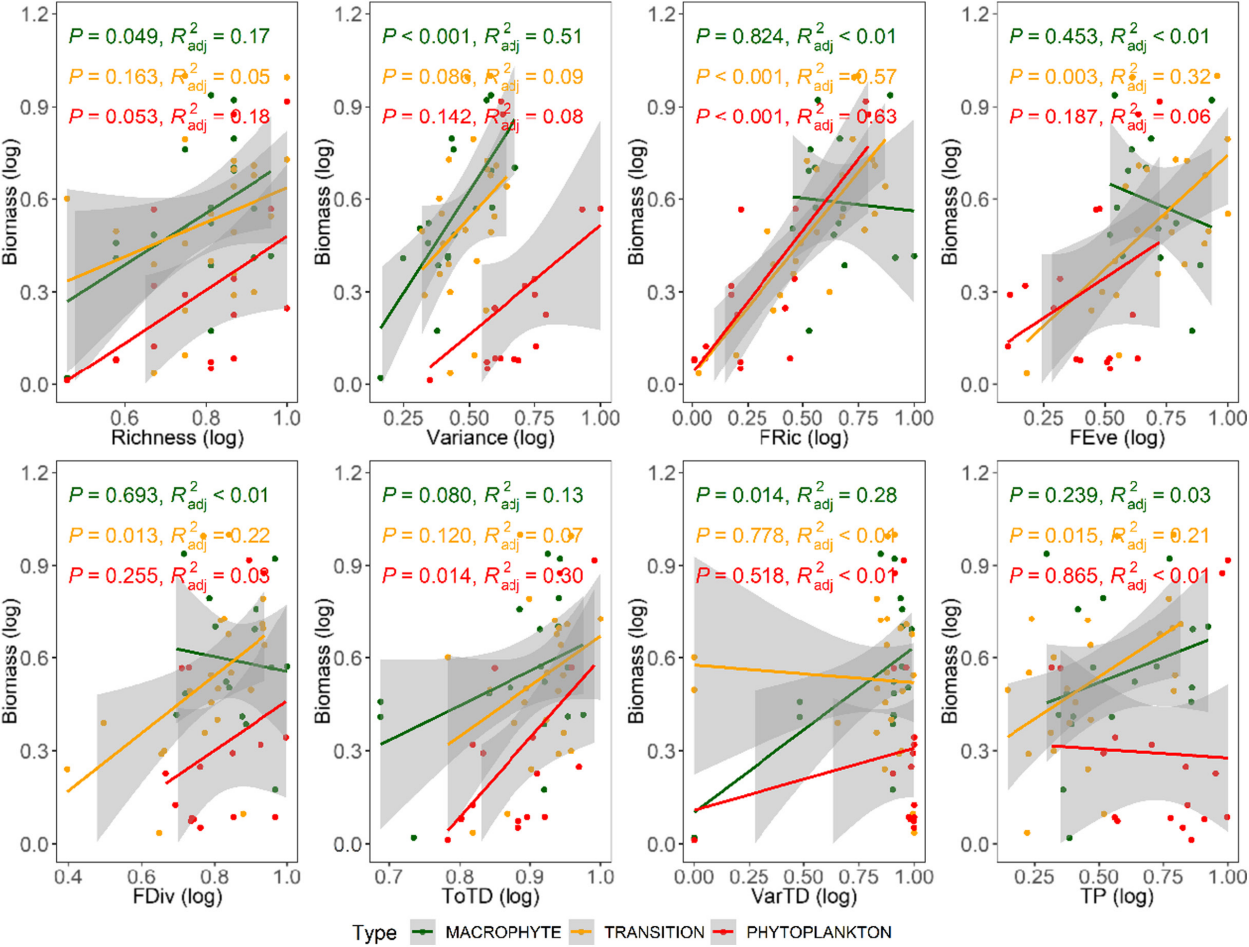
Ordinary least squares (OLS) analysis of the relationship between general biodiversity, functional biodiversity, phylogenetic diversity, and ecosystem functioning across the eutrophic gradient. The black fitted line is from a linear regression. The results of variance for biodiversity are shown by the *R* ^2^ values. Shaded areas show 95% confidence interval of the fit. Only a significant relationship has been presented.

Further, we fitted OLS regression to explore the influence of abiotic factors on macroinvertebrates biomass of each lake type. In *MACROPHYTE* lakes, richness, variance, and VarTD showed a significant positive effect on biomass, where the adjusted *R* ^2^ was 17%, 51%, and 28%, respectively. In *TRANSITION* lakes, FRic, FEve, FDiv, and TP all showed a significant positive effect on biomass, where the adjusted *R* ^2^ was 57%, 32%, 22%, and 21%, respectively. In *PHYTOPLANKTON* lakes, FRic, TOTD showed a significant positive effect on biomass and the adjusted *R* ^2^ were 63% and 30%, respectively (Figure 4).

### 3.4 The effects of biodiversity and abiotic factors on ecosystem functions

We fitted the GLMs to investigate the best predictors of biomass. A total of 55.65% of the variation in macroinvertebrates biomass was accounted for by a model that included FRic and variance (Table 2). A total of 59.90% of the variation in macroinvertebrates biomass was accounted for by a model that included FRic and variance and lake type. A total of 63.59% of the variation in macroinvertebrates biomass was accounted for by a model that included FRic and variance and macrophyte coverage. In isolation, lake type and macrophyte coverage explain 16.51% and 16.46% variation of biomass, respectively.

**TABLE 2:** Summary of the general linear models (GLMs) for the effect of abiotic factors and biodiversity on ecosystem functioning.

Ecosystem functioning	Variable	Estimate	SE	*T*‐value	*p*	VIF
Biomass multiple *R* ^2^: 55.66%	FRic	0.83	0.10	7.99	<.001	1.2
	Variance	0.42	0.18	2.29	.03	1.2

**FIGURE 5:**
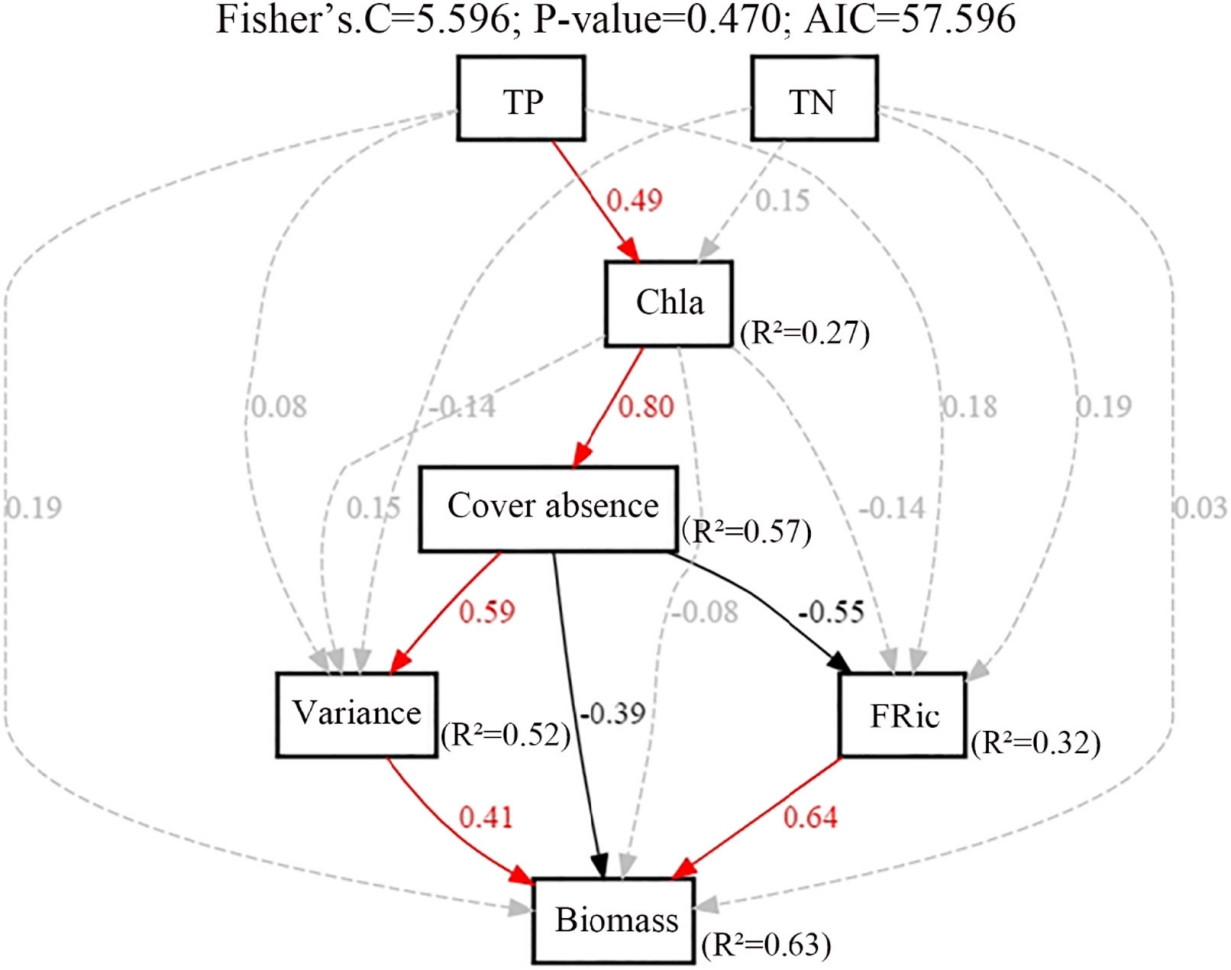
Structural equation models of nutrient, environmental factor, and biodiversity selected by GLMs as predictors of ecosystem functions. Solid red arrows represent positive paths (*p* < .05, piecewise SEM), solid black arrows represent negative paths (*p* < .05, piecewise SEM), and dashed gray arrows represent nonsignificant paths (*p* > .05, piecewise SEM). We have reported the path coefficients as standardized effect sizes. The overall fit of the piecewise SEM was evaluated using the Shipley's test of d‐separation: Fisher's *C* statistic (if *p* > .05, then no paths were missing, and the model was a good fit) and Akaike information criterion (AIC).

Finally, we fitted a piecewise structural equation model to infer how eutrophication (TN，TP，Chl‐a) mediated macroinvertebrates biomass through the dynamics of microinvertebrate diversity in floodplain lakes. The TP variables were found to directly impact on Chl‐a (*β* = 0.49, standardized coefficient), then the extreme Chl‐a concentration induced macrophyte coverage absence through completion (*β* = 0.80, standardized coefficient). The macrophyte coverage absence reduced macroinvertebrates biomass (*β* = −0.39, standardized coefficient) and FRic (*β* = −0.55, standardized coefficient), further strengthens macroinvertebrates community variance (*β* = 0.59, standardized coefficient). The FRic (*β* = 0.64, standardized coefficient) and variance (*β* = 0.41, standardized coefficient) directly influenced the macroinvertebrates biomass (Figure 5).

## 4 DISCUSSION

### 4.1 Environmental factors, macroinvertebrate diversity and ecosystem functions

In our study, significant differences were found among environmental factors, macroinvertebrates species diversity, functional diversity, and biomass between *PHYTOPLANKTON* lakes and other lakes (Table 1) suggesting that even relatively low coverage of macrophytes can make a significant contribution to lake ecosystem function. Absence of macrophytes in *PHYTOPLANKTON* lakes negatively affected macroinvertebrates biomass (Table S5), further strengthens the importance of aquatic macrophytes in Yangtze River lakes (Wang et al., 2006; Zhang et al., 2018). Nutrient loads in lakes primarily neutralize the effect of macrophyte coverage on the macroinvertebrate richness, then enhance eutrophication causing poor macroinvertebrate assemblages (Wright et al., 2002). Eutrophication is reported to have weakened diversity and ecosystem function in many freshwater ecosystems worldwide (Gong & Xie, 2001; Reid et al., 2019). Our study indicated eutrophication could negatively impact on most indices including species diversity, functional diversity, and phylogenetic diversity (Table 1). However, the three diversity facets responded to eutrophication differently in our study lakes. For example, the *PHYTOPLANKTON* lakes were indicative of relatively low Pielou, FEve, FRic, D, AvTD but had similar richness, FDiv, TD, ToTD, and high variance and VarTD when compared with other lakes (Table 1). From the species diversity perspective, the *PHYTOPLANKTON* lakes were found to be moderately diversified but not have an evenly distributed community. Comparatively, a very high occurrence and abundance of some species‐specific macroinvertebrates belonging to Chironomidae and Oligochaeta were observed in the *PHYTOPLANKTON* lakes (Table S4). From the functional diversity perspective, the *PHYTOPLANKTON* lakes were found to be supporting less to ecosystems and the trait showing the unevenly distributed community (Mason et al., 2005). From the phylogenetic diversity perspective, the *PHYTOPLANKTON* lakes were supporting the community with relatively low and uneven phylogenetic diversity (Heino et al., 2007).

It is not surprising, however, that the *PHYTOPLANKTON* lakes were with high variance (the measure of divergence in the community) and VarTD (Table 1). Although the measure of variance is used as an index of species diversity, it strongly correlates with anthropogenic impacts and ecological stress (Stenger‐Kovács et al., 2016). The VarTD, on the other hand, is the measure of irregularities and divergences in the distribution of phylogenetic branch length within a sample. In our study, a high VarTD in the *PHYTOPLANKTON* lakes was resulted from high occurrences of some dominant species belonging to Chironomidae and Oligochaeta.

### 4.2 Relationship between diversity and ecosystem functioning

A strong relationship between macroinvertebrates diversity and ecosystem function was observed as suggested by macroinvertebrates diversity indices in our study (Figure 3). This shows the importance of biodiversity in the maintenance of ecosystem functioning in Yangtze River lakes. Studies suggest that any loss of diversity can lead to a decline in the biomass production of the lake system (Myers et al., 2021; Zimmerman & Cardinale, 2014). We found the effect of diversity on macroinvertebrates biomass varies among three diversity facets. For example, the functional diversity offers greater explanatory power of biomass than species diversity and phylogenetic diversity (Figure 3). The study suggests that higher richness and evenness of functional traits increases food‐use efficiency in most heterogeneous environments as there is no single trait modality exists to dominate the community structure and dynamics (Luiza‐Andrade et al., 2017). Macroinvertebrates community has increased partitioning and utilization ability of the total available resources along with increased functional richness and evenness resulting in increased biomass productivity in the shallow lake system (Cadotte et al., 2011; Luiza‐Andrade et al., 2017). In our study, the greater explanatory power of functional diversity was seen as the indicative of total available resources utilized by macroinvertebrates through niche differentiation or facilitation of resources available in Yangtze River lakes (Figure 3).

Studies suggest that the BEF relationship is largely mediated by abiotic factors and are commonly observed in all sorts of ecosystem including grassland, forest, and intertidal sandflat (Liu et al., 2018; Thrush et al., 2017; Wang et al., 2020). In our study, the BEF relationship was found to be varying along eutrophic gradients suggesting its increasing significance in Yangtze River lakes. A study in an intertidal sandflat demonstrated that the BEF relationship changed along with the sediment nutrient loading (Thrush et al., 2017). In our study, the importance of the BEF relationship is highlighted by spatially dependent shifts in the functional performance, which comprised the variation in macroinvertebrates diversity and biomass under different nutrient loads in Yangtze River lakes. The shifts observed in the functional form of the BEF relationship, for example, from significant to insignificant, and positive to negative relationship (Figures 3 and 4), offer potential insights into the functional resistance and resilience of ecosystems indicating lake ecosystem processes including the recovery under increased nutrient loads or disturbance, as well as the macrophyte dynamics in the lake (Arce et al., 2014).

Complementarity and selection effect are the basis for a positive diversity–community biomass relationship in different ecosystems (Loreau & Hector, 2001). According to the prediction of the niche theory (Hirzel & Lay, 2008), complementarity should be more important in stressful sites, our study is consistent with this view as functional richness, a proxy of niche differentiation, was the main driver of macroinvertebrates biomass in *TRANSITION* and *PHYTOPLANKTON* lakes. While the variance offers the greatest explanation power of macroinvertebrates biomass in *MACROPHYTE* lakes, which proves that selection effect was a pre‐eminent factor in *MACROPHYTE* lakes. It was unexpected, however, that macroinvertebrates biomass in *TRANSITION* lakes was largely dependent on functional diversity (Figure 4) while its diversity index was similar to that of the *MACROPHYTE* lakes (Table 1). The possible reason for this result could be the reduction in the macrophyte coverage, which may have induced the change in macroinvertebrates community assembly, consequently altered the relationship between the biodiversity and biomass.

### 4.3 Abiotic factors mediated links between biodiversity and ecosystem function

Limited roles of abiotic factors on macroinvertebrates biomass including the weaker explanation power of Chl‐a and TP on macroinvertebrates biomass than diversity in our study suggests that ecosystem functioning of Yangtze River lakes is likely to be directly mediated by abiotic factors, and biotic factors act as a linkage between the abiotic factor and ecosystem function (Benkwitt et al., 2020). However, in *TRANSITION* lakes, TP was found to promote macroinvertebrates such as *Corbicula fluminea* biomass by mobilizing the nutrient food resources (Basen et al., 2017). In our study, a high Chl‐a concentration resulted in the low production of macroinvertebrates biomass across all Yangtze River lakes. This could be related to eutrophication, which gradually eliminated sensitive species of gastropods and bivalves (Donohue et al., 2009; Zhang et al., 2019). However, insignificant effect in each lake type, which may result from the high homogeneity of Chl‐a concentration in each lake type. (Thompson et al., 2018).

TP‐induced eutrophication appears to be indirectly impacting on ecosystem functioning of Yangtze River lakes and mobilizing the macroinvertebrates diversity (Figures 4 and 5). For instance, the absence of macrophyte coverage (cover absence) was one of the most influential predictors of macroinvertebrates biomass in the GLMs indicating the enhanced nutrient pathway in the Yangtze River lake ecosystems. The TP primarily promoted phytoplankton growth, and at excessive rates of load, this would lead to the loss of submerged macrophytes due to the shading effect in *PHYTOPLANKTON* lakes (Qin et al., 2006). The coverage absence further mobilized macroinvertebrates diversity both directly and indirectly (FRic and/or variance) followed by macroinvertebrates biomass as revealed by SEMs (Figure 5). Coverage absence reduces FRic and promotes variance, as macrophytes provide diverse niches for species (Yofukuji et al., 2021). Our study provides insight into macroinvertebrates biomass production in Yangtze River lakes, which is highly relied on macrophyte coverage. It has been argued that when the transition of macrophytes types in lakes would occur, the production of consumers including micro‐and‐macroinvertebrates would decrease (Mabidi et al., 2020). First time, we have established transition pathways for TP‐Chl‐a‐Macrophytes‐and‐Macroinvertebrate biomass in Yangtze River lakes (Figure 5), an indicative of the ecosystem health of the middle and lower reaches of the Yangtze basin.

## 5 CONCLUSIONS

We have revealed significant changes in the environmental drivers, macroinvertebrates diversity, and biomass along the eutrophication gradients of the middle and lower reaches of the Yangtze River basin (China). Our study suggests that the nutrient enrichments in these lakes during the early stage could weaken the effect of macrophyte coverage and reduce macroinvertebrates diversity (except Variance and VarTD) and biomass. However, increased nutrient enrichments in the lake system over a longer period can lead to overwhelming impacts on biomass. We found that the functional diversity could offer a better explanatory power in the ecosystem function variation, which is more than species diversity and phylogenetic diversity. The BEF relationship is dependent on the spatial scale measurement, as well as the condition of the local environment, as we observed a significant difference in the BEF relationship among different types of Yangtze River lakes. Furthermore, the BEF relationship varies along eutrophication gradients, and the niche differentiation and the species dominance both can drive macroinvertebrates biomass production in shallow lowland Yangtze River lowland shallow systems. Eutrophication could not only affect the BEF relationship directly by altering community composition but also influence indirectly by changing the macrophyte coverage. Hence, the presence of macrophyte coverage is of greater significance in Yangtze River lakes that are exposed to prolonged eutrophication. Our study highlights the need for urgent countermeasures to reduce nutrient inputs and the restoration of the submerged macrophyte community, as these actions are indispensable to maintain biodiversity and ecosystem function, as well as to sustain lake ecosystem services.

## AUTHOR CONTRIBUTIONS

**Yongjiu Cai:** Conceptualization (equal); funding acquisition (lead); investigation (equal); writing – original draft (equal); writing – review and editing (lead). **Rui Dong:** Data curation (equal); formal analysis (equal); writing – original draft (equal); writing – review and editing (equal). **Giri Kattel:** Conceptualization (equal); writing – original draft (equal); writing – review and editing (equal). **You Zhang:** Investigation (equal); writing – review and editing (equal). **Kai Peng:** Investigation (equal); writing – review and editing (equal). **Zhijun Gong:** Conceptualization (equal); supervision (lead); writing – review and editing (equal).

## CONFLICT OF INTEREST

The authors declare no conflict of interest.

## Supporting information


Appendix S1:

## Data Availability

The data that support the findings of this study are available from the corresponding author upon reasonable request.
