# Corrigendum

**DOI:** 10.1002/ece3.9815

**Published:** 2023-02-19

In the recent article by Cai et al. (2023), the authors would like to add the following ORCID ID for Giri Kattel:

https://orcid.org/0000‐0002‐8348‐6477
