# Supplementary material for: Macroinvertebrate diversity and ecosystem functioning across the eutrophication gradients of the middle and lower reaches of Yangtze River lakes (China)
Source: Ecol Evol. 2023 Jan 18;13(1):e9751. doi: 10.1002/ece3.9751 (PMC9846310; doi:10.1002/ece3.9751)
Supplement: Supplementary file 1 — Appendix S1: [file ECE3-13-e9751-s001.docx]

**Appendix 1**

# Table S1. Limnological parameters of the three different types, *MACROPHYTE*, *TRANSITION*, *PHYTOPLANKTON*, of lakes in the middle and lower Yangtze basin.

| Lake type | Lakes | Abbreviation | Longitude | Latitude | Water area (km^2^) | Max water depth (m) | Mean water depth (m) |
| --- | --- | --- | --- | --- | --- | --- | --- |
| MACROPHYTE lakes | Hong Lake | HH | 113.12' -113°26'E | 29°40' -29°58'N | 344 | 2.3 | 1.4 |
|  | Chi Lake | CH | 115°37' -115°44'E | 29°44' -29°50'N | 51 | 3.6 | 2.5 |
|  | Daye Lake | DY | 115°02' -115°11'E | 30°04' -30°08'N | 69 | 2.3 | 1.92 |
|  | Baoying Lake | BY | 120°48' -120°52'E | 31°10' -31°14'N | 43 | 2.2 | 1.1 |
| TRANSITION lakes | Anle Lake | AL | 112°06' -112°12'E | 28°48' -28°50'N | 30 | 4.6 | 4.2 |
|  | Junshan Lake | JS | 116°15' -116°28'E | 28°24' -28°38'N | 160 | 6.4 | 4.0 |
|  | Zhu Lake | ZH | 116°34' -116°45'E | 29°04' -29°12'N | 64 | 7.4 | 4.9 |
|  | Kuilei Lake | KL | 120°50' -120°52'E | 31°23' -31°26'N | 7 | 7.5 | 1.5 |
| PHYTOPLANKTON lakes | Beimin Lake | BM | 111°51' -111°55'E | 29°42' -29°46'N | 14 | 3.0 | 2.8 |
|  | Yueyang South Lake | YY | 113°05' -113°11'E | 29°27' -29°31'N | 10 | 5.7 | 5.4 |
|  | Chenjia Lake | CJ | 116°21' -116°24'E | 29°38' -29°41'N | 22 | 6.2 | 5.5 |
|  | Ge Lake | GH | 119°44' -119°53'E | 31°29' -31°42'N | 144 | 1.9 | 1.2 |

**Table S2.** Sixty functional categories of 10 traits used to calculate observed degree of specialization of taxa within communities (Bis and Usseglio-Polatera, 2005; Mondy and Usseglio-Polatera, 2014)

| Traits | categories | |  | Traits | categories | |
| --- | --- | --- | --- | --- | --- | --- |
| Maximal size | 1 | ≤0.25cm |  | Respiration | 1 | tegument |
|  | 2 | 0.25-0.5cm |  |  | 2 | gill |
|  | 3 | 0.5-1cm |  |  | 3 | plastron |
|  | 4 | 1-2cm |  |  | 4 | Spiracle (aerial) |
|  | 5 | 2-4cm |  |  | 5 | hydrostatic vesicle (aerial) |
|  | 6 | 4-8cm |  | Locomotion and substrate relation | 1 | flier |
|  | 7 | >8cm |  |  | 2 | surface swimmer |
| Life cycle duration | 1 | ≤1 year |  |  | 3 | full water swimmer |
|  | 2 | >1 year |  |  | 4 | crawler |
| Aquatic stages | 1 | egg |  |  | 5 | Burrower (epibenthic) |
|  | 2 | larva |  |  | 6 | Interstitial (epibenthic) |
|  | 3 | pupa |  |  | 7 | temporarily attached |
|  | 4 | adult |  |  | 8 | permanently attached |
| Reproduction | 1 | ovoviviparity |  | Food | 1 | fine sediment + microorganisms |
|  | 2 | isolated eggs, free |  |  | 2 | detritus<1mm |
|  | 3 | isolated eggs, cemented |  |  | 3 | plant detritus>1mm |
|  | 4 | clutches,cemented or fixed |  |  | 4 | living microphytes |
|  | 5 | clutches,free |  |  | 5 | living macrophytes |
|  | 6 | clutches,in vegetation |  |  | 6 | dead animal>1mm |
|  | 7 | clutches, terrestrial |  |  | 7 | living microinvertebrates |
|  | 8 | asexual reproduction |  |  | 8 | living macroinvertebrates |
| Dissemination | 1 | aquatic passive |  |  | 9 | vertebrates |
|  | 2 | aquatic active |  | Feeding habits | 1 | absorber |
|  | 3 | aerial passive |  |  | 2 | deposit feeder |
|  | 4 | aerial active |  |  | 3 | shredder |
| Resistance form | 1 | eggs, statoblasts |  |  | 4 | scraper |
|  | 2 | cocoons |  |  | 5 | filter feeder |
|  | 3 | cells against desiccation |  |  | 6 | piercer (plants or animals) |
|  | 4 | diapause or dormancy |  |  | 7 | predator (carver/engulfer/swallower) |
|  | 5 | none |  |  | 8 | parasite |

**References:**

Bis, B., Usseglio-Polatera, P., 2005. The EU-STAR: Species traits analysis: The application of species trait analysis to the establishment of reference conditions and the assessment of Ecological Status for the implementation of the Water Framework Directive (Deliverable N2).

Mondy, C.P., Usseglio-Polatera, P., 2014. Using fuzzy-coded traits to elucidate the non-random role of anthropogenic stress in the functional homogenisation of invertebrate assemblages. Freshwater Biology 59, 584-600.

**Table S3.** Environmental parameters of *MACROPHYTE*, *TRANSITION* and *PHYTOPLANKTON* lakes.

| Parameters | *MACROPHYTE* | *TRANSITION* | *PHYTOPLANKTON* | *P* |
| --- | --- | --- | --- | --- |
| WT (℃) | 21.0±1.0 | 22.8±1.3 | 21.9±1.3 | 0.543 |
| SD (m) | 0.99±0.12 | 1.31±0.17 | 0.41±0.05 | <0.001 |
| pH | 8.5±0.2 | 7.8±0.1 | 8.0±0.2 | 0.001 |
| DO (mg/L) | 8.1±1.9 | 8.6±0.4 | 7.7±0.3 | 0.07 |
| Chl-a (ug/L) | 4.18±0.47 | 4.39±0.58 | 24.97±3.71 | <0.001 |
| TN (mg/L) | 1.23±0.132 | 1.01±0.13 | 1.82±0.19 | 0.002 |
| TP (mg/L) | 0.032±0.004 | 0.041±0.004 | 0.103±0.012 | <0.001 |
| Macrophyte cover class | 4.17±0.60 | 1.83±0.80 | 0±0 | <0.001 |

**Table S4.** Abundance (ind/m^2^) of dominant macroinvertebrate species in different type of lakes.

| Class | Species | *MACROPHYTE* | *TRANSITION* | *PHYTOPLANKTON* |
| --- | --- | --- | --- | --- |
| Gastropoda | *Bellamya aeruginosa* | 63.59±13.21 | 63.33±19.62 |  |
|  | *Parafossarulus striatulus* | 63.74±15.69 |  |  |
|  | *Alocinma longicornis* | 86.61±30.19 | 43.00±19.57 |  |
| Bivalvia | *Corbicula fluminea* |  | 97.68±32.21 |  |
| Insecta | *Propsilocerus akamusi* | 66.67±48.17 |  |  |
|  | *Chironomus* *flaviplumus* | 79.57±28.88 |  | 325.94±136.29 |
|  | *Microchironomus tabarui* |  |  | 160.65±63.29 |
| Polychaeta | *Nephtys oligobranchia* |  | 60.33±19.72 |  |
| Oligochaeta | *Branchiura sowerbyi* | 19.78±5.54 | 41.67±9.51 | 133.94±32.47 |
|  | *Limnodrilus hoffmeisteri* |  | 63.67±21.49 | 1242.18±599.85 |

**Table S5**. Cumulative contributions of most influential species to community structure difference among three types of lakes，*MACROPHYTE*, *TRANSITION* and *PHYTOPLANKTON*.

| Comparison | Species | Cumulative contributions |
| --- | --- | --- |
| MACROPHYTE  vs  TRANSITION | *Corbicula fluminea* | 12.66% |
|  | *Alocinma longicornis* | 23.81% |
|  | *Bellamya aeruginosa* | 33.29% |
|  | *Chironomus flaviplumus* | 42.33% |
|  | *Nephtys oligobranchia* | 51.21% |
|  | *Parafossarulus striatulus* | 58.81% |
|  | *Limnodrilus hoffmeisteri* | 65.86% |
|  | *Propsilocerus akamusi* | 72.66% |
| MACROPHYTE  vs  PHYTOPLANKTON | *Limnodrilus hoffmeisteri* | 27.83% |
|  | *Chironomus flaviplumus* | 46.36% |
|  | *Microchironomus tabarui* | 58.39% |
|  | *Branchiura sowerbyi* | 63.85% |
|  | *Propsilocerus akamusi* | 69.26% |
|  | *Alocinma longicornis* | 74.19% |
| TRANSITION  vs  PHYTOPLANKTON | *Limnodrilus hoffmeisteri* | 27.75% |
|  | *Chironomus flaviplumus* | 44.68% |
|  | *Microchironomus tabarui* | 56.28% |
|  | *Corbicula fluminea* | 64.19% |
|  | *Branchiura sowerbyi* | 69.41% |
|  | *Propsilocerus akamusi* | 73.58% |

**Table S6.** Macroinvertebrates abundance (ind/m^2^) of the 12 lakes belonging to different lake type

| Taxa | *MACROPHYTE* | | | |  | *TRANSITION* | | | |  | *PHYTOPLANKTON* | | | |
| --- | --- | --- | --- | --- | --- | --- | --- | --- | --- | --- | --- | --- | --- | --- |
|  | HH | CH | DY | BY |  | AL | JS | ZH | KL |  | BM | YY | CJ | GH |
| **Oligochaeta** |  |  |  |  |  |  |  |  |  |  |  |  |  |  |
| *Aulodrilus pluriseta* | 0.000 | 0.000 | 0.000 | 0.000 |  | 0.000 | 13.333 | 0.000 | 0.000 |  | 0.000 | 0.000 | 0.000 | 0.000 |
| *Branchiodrilus hortensis* | 5.333 | 0.000 | 0.000 | 0.000 |  | 0.000 | 0.000 | 0.000 | 0.000 |  | 0.000 | 36.000 | 0.000 | 0.000 |
| *Branchiura sowerbyi* | 30.000 | 22.667 | 26.667 | 8.333 |  | 53.333 | 64.000 | 29.333 | 20.000 |  | 164.000 | 244.000 | 80.000 | 67.500 |
| *Limnodrilus grandisetosus* | 0.000 | 2.667 | 0.000 | 0.000 |  | 5.333 | 0.000 | 0.000 | 0.000 |  | 38.000 | 0.000 | 0.000 | 0.000 |
| *Limnodrilus hoffmeisteri* | 40.000 | 9.333 | 10.667 | 0.000 |  | 93.333 | 0.000 | 1.333 | 160.000 |  | 78.000 | 4750.000 | 0.000 | 300.833 |
| *Rhyacodrilus sinicus* | 0.000 | 0.000 | 5.333 | 0.000 |  | 26.667 | 0.000 | 0.000 | 0.000 |  | 92.000 | 560.000 | 0.000 | 0.000 |
| **Insecta** |  |  |  |  |  |  |  |  |  |  |  |  |  |  |
| *Ablabesmyia* sp. | 0.000 | 0.000 | 0.000 | 0.000 |  | 0.000 | 0.000 | 2.667 | 0.000 |  | 0.000 | 0.000 | 0.000 | 0.000 |
| *Chironomus flaviplumus* | 5.333 | 122.667 | 117.333 | 54.722 |  | 106.667 | 0.000 | 0.000 | 0.000 |  | 0.000 | 152.000 | 1469.333 | 87.500 |
| *Cladopelma* sp*.* | 0.000 | 0.000 | 0.000 | 0.000 |  | 0.000 | 0.000 | 0.000 | 6.667 |  | 0.000 | 0.000 | 0.000 | 0.000 |
| *Clinotanypus* sp. | 5.333 | 0.000 | 0.000 | 0.000 |  | 24.000 | 10.667 | 33.333 | 6.667 |  | 110.000 | 18.000 | 21.333 | 0.000 |
| *Cricotopus sylvestris* | 5.333 | 0.000 | 0.000 | 9.444 |  | 0.000 | 0.000 | 0.000 | 0.000 |  | 0.000 | 0.000 | 0.000 | 0.000 |
| *Cryptochironomus digitatus* | 0.000 | 0.000 | 0.000 | 0.000 |  | 0.000 | 0.000 | 0.000 | 0.000 |  | 0.000 | 0.000 | 0.000 | 10.000 |
| *Cryptochironomus* sp. | 0.000 | 0.000 | 0.000 | 0.000 |  | 0.000 | 0.000 | 0.000 | 53.333 |  | 32.000 | 0.000 | 0.000 | 0.000 |
| *Crytotendipes* sp. | 0.000 | 0.000 | 0.000 | 0.000 |  | 0.000 | 0.000 | 0.000 | 6.667 |  | 0.000 | 0.000 | 0.000 | 0.000 |
| *Dicrotendipus lobifer* | 0.000 | 0.000 | 0.000 | 8.333 |  | 0.000 | 0.000 | 0.000 | 0.000 |  | 0.000 | 0.000 | 0.000 | 0.000 |
| *Glyptotendipes cauliginellus* | 0.000 | 2.667 | 5.333 | 0.000 |  | 0.000 | 0.000 | 0.000 | 0.000 |  | 0.000 | 0.000 | 0.000 | 0.000 |
| *Glyptotendipes* sp. | 0.000 | 0.000 | 0.000 | 0.000 |  | 0.000 | 0.000 | 0.000 | 13.333 |  | 0.000 | 0.000 | 0.000 | 0.000 |
| *Harnischia fuscimana* | 0.000 | 0.000 | 0.000 | 0.000 |  | 0.000 | 0.000 | 0.000 | 0.000 |  | 0.000 | 0.000 | 0.000 | 1.667 |
| *Microchironomus tabarui* | 0.000 | 0.000 | 26.667 | 0.000 |  | 10.667 | 0.000 | 2.667 | 73.333 |  | 114.000 | 0.000 | 0.000 | 379.167 |
| *Microchironomus tener* | 0.000 | 0.000 | 0.000 | 0.000 |  | 0.000 | 0.000 | 0.000 | 0.000 |  | 0.000 | 0.000 | 0.000 | 8.333 |
| *Polypedilum nubeculosum* | 0.000 | 0.000 | 0.000 | 29.167 |  | 0.000 | 0.000 | 0.000 | 0.000 |  | 0.000 | 0.000 | 0.000 | 0.000 |
| *Polypedilum scalaenum* | 0.000 | 0.000 | 0.000 | 0.000 |  | 0.000 | 0.000 | 0.000 | 0.000 |  | 0.000 | 0.000 | 0.000 | 6.667 |
| *Polypedilum* sp. | 0.000 | 2.667 | 5.333 | 0.000 |  | 0.000 | 2.667 | 0.000 | 53.333 |  | 0.000 | 0.000 | 0.000 | 0.000 |
| *Procladius choreus* | 0.000 | 0.000 | 0.000 | 0.000 |  | 0.000 | 2.667 | 6.667 | 0.000 |  | 120.000 | 44.000 | 10.667 | 0.000 |
| *Procladius* sp. | 0.000 | 0.000 | 0.000 | 0.000 |  | 0.000 | 0.000 | 0.000 | 53.333 |  | 0.000 | 0.000 | 0.000 | 0.000 |
| *Propsilocerus akamusi* | 16.000 | 0.000 | 384.000 | 0.000 |  | 0.000 | 0.000 | 0.000 | 120.000 |  | 206.000 | 8.000 | 32.000 | 0.000 |
| *Tanypus chinensis* | 0.000 | 0.000 | 0.000 | 0.000 |  | 29.333 | 0.000 | 6.667 | 0.000 |  | 34.000 | 150.000 | 10.667 | 8.333 |
| **Bivalvia** |  |  |  |  |  |  |  |  |  |  |  |  |  |  |
| *Acuticosta* sp*.* | 0.000 | 0.000 | 0.000 | 0.000 |  | 4.000 | 0.000 | 0.000 | 0.000 |  | 0.000 | 0.000 | 0.000 | 0.000 |
| *Corbicula fluminea* | 0.000 | 0.000 | 0.000 | 0.000 |  | 34.667 | 266.667 | 49.333 | 40.000 |  | 160.000 | 0.000 | 5.333 | 0.000 |
| *Lamprotula* sp*.* | 0.000 | 0.000 | 0.000 | 0.000 |  | 0.000 | 0.000 | 0.000 | 0.000 |  | 4.000 | 0.000 | 0.000 | 0.000 |
| *Lanceolaria gladiola* | 0.000 | 0.000 | 0.000 | 0.000 |  | 0.000 | 0.000 | 0.000 | 0.000 |  | 4.000 | 0.000 | 0.000 | 0.000 |
| *Limnoperna fortunei* | 0.000 | 0.000 | 0.000 | 0.000 |  | 26.667 | 8.000 | 0.000 | 0.000 |  | 0.000 | 0.000 | 0.000 | 0.000 |
| *Unio douglasiae* | 0.000 | 0.000 | 0.000 | 0.000 |  | 1.333 | 2.667 | 0.000 | 0.000 |  | 0.000 | 0.000 | 0.000 | 0.000 |
| **Gastropoda** |  |  |  |  |  |  |  |  |  |  |  |  |  |  |
| *Alocinma longicornis* | 53.333 | 8.000 | 272.000 | 89.167 |  | 25.333 | 0.000 | 0.000 | 146.667 |  | 0.000 | 0.000 | 0.000 | 0.000 |
| *Bellamya aeruginosa* | 42.667 | 85.333 | 69.333 | 49.444 |  | 65.333 | 18.667 | 2.667 | 166.667 |  | 48.000 | 0.000 | 0.000 | 0.000 |
| *Gyraulus convexiusculus* | 0.000 | 2.667 | 0.000 | 0.000 |  | 0.000 | 0.000 | 0.000 | 0.000 |  | 0.000 | 0.000 | 0.000 | 0.000 |
| *Hippeutis umbilicalis* | 42.667 | 12.000 | 0.000 | 0.000 |  | 0.000 | 0.000 | 0.000 | 0.000 |  | 0.000 | 0.000 | 0.000 | 0.000 |
| *Parafossarulus eximius* | 0.000 | 2.667 | 0.000 | 0.000 |  | 13.333 | 0.000 | 0.000 | 6.667 |  | 0.000 | 0.000 | 0.000 | 0.000 |
| *Parafossarulus striatulus* | 21.333 | 72.000 | 69.333 | 73.889 |  | 5.333 | 0.000 | 0.000 | 6.667 |  | 0.000 | 0.000 | 0.000 | 0.000 |
| *Radix swinhoei* | 10.667 | 30.667 | 0.000 | 0.000 |  | 0.000 | 0.000 | 0.000 | 0.000 |  | 0.000 | 0.000 | 0.000 | 0.000 |
| *Rivularia auriculata* | 0.000 | 0.000 | 0.000 | 0.000 |  | 0.000 | 10.667 | 0.000 | 0.000 |  | 0.000 | 0.000 | 0.000 | 0.000 |
| *Semisulcospira cancelata* | 0.000 | 0.000 | 0.000 | 0.000 |  | 5.333 | 0.000 | 0.000 | 0.000 |  | 36.000 | 0.000 | 0.000 | 0.000 |
| *Stenothyra glabra* | 5.333 | 0.000 | 0.000 | 0.000 |  | 0.000 | 8.000 | 0.000 | 0.000 |  | 0.000 | 0.000 | 0.000 | 0.000 |
| **Miscellaneous** |  |  |  |  |  |  |  |  |  |  |  |  |  |  |
| *Exopalaemon modestus* | 0.000 | 4.000 | 0.000 | 0.000 |  | 0.000 | 0.000 | 0.000 | 0.000 |  | 0.000 | 0.000 | 0.000 | 0.000 |
| *Gammarus sp.* | 0.000 | 0.000 | 0.000 | 1.667 |  | 0.000 | 0.000 | 0.000 | 0.000 |  | 0.000 | 0.000 | 0.000 | 0.000 |
| *Neocaridina denticulata* | 5.333 | 0.000 | 0.000 | 0.000 |  | 0.000 | 0.000 | 0.000 | 0.000 |  | 0.000 | 0.000 | 0.000 | 0.000 |
| *Nephtys oligobranchia* | 0.000 | 0.000 | 0.000 | 0.000 |  | 0.000 | 176.000 | 65.333 | 0.000 |  | 0.000 | 0.000 | 0.000 | 0.000 |
| Glossiphoniidae sp. | 0.000 | 0.000 | 0.000 | 0.000 |  | 2.667 | 0.000 | 0.000 | 0.000 |  | 0.000 | 0.000 | 0.000 | 0.000 |
| *Glossiphonia complanata* | 0.000 | 5.333 | 0.000 | 1.667 |  | 0.000 | 0.000 | 2.667 | 0.000 |  | 24.000 | 128.000 | 0.000 | 0.000 |
| *Helobdella fusca* | 0.000 | 0.000 | 5.333 | 0.000 |  | 0.000 | 0.000 | 0.000 | 0.000 |  | 0.000 | 0.000 | 0.000 | 0.000 |
| *Erpobdella octoculata* | 0.000 | 0.000 | 0.000 | 0.000 |  | 0.000 | 0.000 | 2.667 | 0.000 |  | 0.000 | 2.000 | 0.000 | 0.000 |

**Table S7**. Macroinvertebrates biomass(g/m^2^) of the 12 lakes belonging to different lake type

| Taxa | *MACROPHYTE* | | | |  | *TRANSITION* | | | |  | *PHYTOPLANKTON* | | | |
| --- | --- | --- | --- | --- | --- | --- | --- | --- | --- | --- | --- | --- | --- | --- |
|  | HH | CH | DY | BY |  | AL | JS | ZH | KL |  | BM | YY | CJ | GH |
| **Oligochaeta** |  |  |  |  |  |  |  |  |  |  |  |  |  |  |
| *Aulodrilus pluriseta* | 0.000 | 0.000 | 0.000 | 0.000 |  | 0.000 | 0.005 | 0.000 | 0.000 |  | 0.000 | 0.000 | 0.000 | 0.000 |
| *Branchiodrilus hortensis* | 0.005 | 0.000 | 0.000 | 0.000 |  | 0.000 | 0.000 | 0.000 | 0.000 |  | 0.000 | 0.066 | 0.000 | 0.000 |
| *Branchiura sowerbyi* | 0.594 | 0.250 | 0.226 | 8.333 |  | 0.879 | 0.615 | 0.517 | 0.041 |  | 1.964 | 5.583 | 2.415 | 0.278 |
| *Limnodrilus grandisetosus* | 0.000 | 0.003 | 0.000 | 0.000 |  | 0.008 | 0.000 | 0.000 | 0.000 |  | 0.064 | 0.000 | 0.000 | 0.000 |
| *Limnodrilus hoffmeisteri* | 0.089 | 0.004 | 0.020 | 0.000 |  | 0.167 | 0.000 | 0.005 | 0.239 |  | 0.155 | 10.199 | 0.000 | 0.289 |
| *Rhyacodrilus sinicus* | 0.000 | 0.000 | 0.003 | 0.000 |  | 0.013 | 0.000 | 0.000 | 0.000 |  | 0.077 | 0.274 | 0.000 | 0.000 |
| **Insecta** |  |  |  |  |  |  |  |  |  |  |  |  |  |  |
| *Ablabesmyia* sp. | 0.000 | 0.000 | 0.000 | 0.000 |  | 0.000 | 0.000 | 0.001 | 0.000 |  | 0.000 | 0.000 | 0.000 | 0.000 |
| *Chironomus flaviplumus* | 0.012 | 0.260 | 0.379 | 0.098 |  | 0.578 | 0.000 | 0.000 | 0.000 |  | 0.000 | 2.163 | 9.048 | 0.392 |
| *Cladopelma* sp*.* | 0.000 | 0.000 | 0.000 | 0.000 |  | 0.000 | 0.000 | 0.000 | 0.001 |  | 0.000 | 0.000 | 0.000 | 0.000 |
| *Clinotanypus* sp. | 0.013 | 0.000 | 0.000 | 0.000 |  | 0.037 | 0.022 | 0.052 | 0.025 |  | 0.236 | 0.048 | 0.030 | 0.000 |
| *Cricotopus sylvestris* | 0.005 | 0.000 | 0.000 | 0.003 |  | 0.000 | 0.000 | 0.000 | 0.000 |  | 0.000 | 0.000 | 0.000 | 0.000 |
| *Cryptochironomus digitatus* | 0.000 | 0.000 | 0.000 | 0.000 |  | 0.000 | 0.000 | 0.000 | 0.000 |  | 0.000 | 0.000 | 0.000 | 0.005 |
| *Cryptochironomus* sp. | 0.000 | 0.000 | 0.000 | 0.000 |  | 0.000 | 0.000 | 0.000 | 0.033 |  | 0.139 | 0.000 | 0.000 | 0.000 |
| *Crytotendipes* sp. | 0.000 | 0.000 | 0.000 | 0.000 |  | 0.000 | 0.000 | 0.000 | 0.026 |  | 0.000 | 0.000 | 0.000 | 0.000 |
| *Dicrotendipus lobifer* | 0.000 | 0.000 | 0.000 | 0.003 |  | 0.000 | 0.000 | 0.000 | 0.000 |  | 0.000 | 0.000 | 0.000 | 0.000 |
| *Glyptotendipes cauliginellus* | 0.000 | 0.006 | 0.020 | 0.000 |  | 0.000 | 0.000 | 0.000 | 0.000 |  | 0.000 | 0.000 | 0.000 | 0.000 |
| *Glyptotendipes* sp. | 0.000 | 0.000 | 0.000 | 0.000 |  | 0.000 | 0.000 | 0.000 | 0.002 |  | 0.000 | 0.000 | 0.000 | 0.000 |
| *Harnischia fuscimana* | 0.000 | 0.000 | 0.000 | 0.000 |  | 0.000 | 0.000 | 0.000 | 0.000 |  | 0.000 | 0.000 | 0.000 | 0.001 |
| *Microchironomus tabarui* | 0.000 | 0.000 | 0.010 | 0.000 |  | 0.003 | 0.000 | 0.001 | 0.015 |  | 0.090 | 0.000 | 0.000 | 0.456 |
| *Microchironomus tener* | 0.000 | 0.000 | 0.000 | 0.000 |  | 0.000 | 0.000 | 0.000 | 0.000 |  | 0.000 | 0.000 | 0.000 | 0.004 |
| *Polypedilum nubeculosum* | 0.000 | 0.000 | 0.000 | 0.025 |  | 0.000 | 0.000 | 0.000 | 0.000 |  | 0.000 | 0.000 | 0.000 | 0.000 |
| *Polypedilum scalaenum* | 0.000 | 0.000 | 0.000 | 0.000 |  | 0.000 | 0.000 | 0.000 | 0.000 |  | 0.000 | 0.000 | 0.000 | 0.006 |
| *Polypedilum* sp. | 0.000 | 0.001 | 0.005 | 0.000 |  | 0.000 | 0.006 | 0.000 | 0.024 |  | 0.000 | 0.000 | 0.000 | 0.000 |
| *Procladius choreus* | 0.000 | 0.000 | 0.000 | 0.000 |  | 0.000 | 0.001 | 0.004 | 0.000 |  | 0.130 | 0.085 | 0.020 | 0.000 |
| *Procladius* sp. | 0.000 | 0.000 | 0.000 | 0.000 |  | 0.000 | 0.000 | 0.000 | 0.092 |  | 0.000 | 0.000 | 0.000 | 0.000 |
| *Propsilocerus akamusi* | 0.303 | 0.000 | 7.398 | 0.000 |  | 0.000 | 0.000 | 0.000 | 1.275 |  | 3.214 | 0.258 | 0.181 | 0.000 |
| *Tanypus chinensis* | 0.000 | 0.000 | 0.000 | 0.000 |  | 0.065 | 0.000 | 0.016 | 0.000 |  | 0.114 | 1.322 | 0.064 | 0.152 |
| **Bivalvia** |  |  |  |  |  |  |  |  |  |  |  |  |  |  |
| *Acuticosta* sp*.* | 0.000 | 0.000 | 0.000 | 0.000 |  | 18.174 | 0.000 | 0.000 | 0.000 |  | 0.000 | 0.000 | 0.000 | 0.000 |
| *Corbicula fluminea* | 0.000 | 0.000 | 0.000 | 0.000 |  | 64.834 | 113.753 | 127.820 | 70.473 |  | 7.023 | 0.000 | 4.332 | 0.000 |
| *Lamprotula* sp*.* | 0.000 | 0.000 | 0.000 | 0.000 |  | 0.000 | 0.000 | 0.000 | 0.000 |  | 0.000 | 0.000 | 0.000 | 0.000 |
| *Lanceolaria gladiola* | 0.000 | 0.000 | 0.000 | 0.000 |  | 0.000 | 0.000 | 0.000 | 0.000 |  | 0.000 | 0.000 | 0.000 | 0.000 |
| *Limnoperna fortunei* | 0.000 | 0.000 | 0.000 | 0.000 |  | 12.805 | 1.371 | 0.000 | 0.000 |  | 0.000 | 0.000 | 0.000 | 0.000 |
| *Unio douglasiae* | 0.000 | 0.000 | 0.000 | 0.000 |  | 9.720 | 16.741 | 0.000 | 0.000 |  | 0.000 | 0.000 | 0.000 | 0.000 |
| **Gastropoda** |  |  |  |  |  |  |  |  |  |  |  |  |  |  |
| *Alocinma longicornis* | 5.537 | 0.773 | 40.466 | 11.179 |  | 4.684 | 0.000 | 0.000 | 16.813 |  | 0.000 | 0.000 | 0.000 | 0.000 |
| *Bellamya aeruginosa* | 87.342 | 107.084 | 176.426 | 67.619 |  | 107.734 | 6.770 | 0.057 | 176.467 |  | 46.426 | 0.000 | 0.000 | 0.000 |
| *Gyraulus convexiusculus* | 0.000 | 0.053 | 0.000 | 0.000 |  | 0.000 | 0.000 | 0.000 | 0.000 |  | 0.000 | 0.000 | 0.000 | 0.000 |
| *Hippeutis umbilicalis* | 0.800 | 0.277 | 0.000 | 0.000 |  | 0.000 | 0.000 | 0.000 | 0.000 |  | 0.000 | 0.000 | 0.000 | 0.000 |
| *Parafossarulus eximius* | 0.000 | 2.667 | 0.000 | 0.000 |  | 11.467 | 0.000 | 0.000 | 4.733 |  | 0.000 | 0.000 | 0.000 | 0.000 |
| *Parafossarulus striatulus* | 4.001 | 5.663 | 18.282 | 13.677 |  | 1.344 | 0.000 | 0.000 | 0.800 |  | 0.000 | 0.000 | 0.000 | 0.000 |
| *Radix swinhoei* | 1.282 | 1.540 | 0.000 | 0.000 |  | 0.000 | 0.000 | 0.000 | 0.000 |  | 0.000 | 0.000 | 0.000 | 0.000 |
| *Rivularia auriculata* | 0.000 | 0.000 | 0.000 | 0.000 |  | 0.000 | 26.317 | 0.000 | 0.000 |  | 0.000 | 0.000 | 0.000 | 0.000 |
| *Semisulcospira cancelata* | 0.000 | 0.000 | 0.000 | 0.000 |  | 0.251 | 0.000 | 0.000 | 0.000 |  | 2.217 | 0.000 | 0.000 | 0.000 |
| *Stenothyra glabra* | 12.157 | 0.000 | 0.000 | 0.000 |  | 0.000 | 0.070 | 0.000 | 0.000 |  | 0.000 | 0.000 | 0.000 | 0.000 |
| **Miscellaneous** |  |  |  |  |  |  |  |  |  |  |  |  |  |  |
| *Exopalaemon modestus* | 0.000 | 1.885 | 0.000 | 0.000 |  | 0.000 | 0.000 | 0.000 | 0.000 |  | 0.000 | 0.000 | 0.000 | 0.000 |
| *Gammarus sp.* | 0.000 | 0.000 | 0.000 | 0.003 |  | 0.000 | 0.000 | 0.000 | 0.000 |  | 0.000 | 0.000 | 0.000 | 0.000 |
| *Neocaridina denticulata* | 0.274 | 0.000 | 0.000 | 0.000 |  | 0.000 | 0.000 | 0.000 | 0.000 |  | 0.000 | 0.000 | 0.000 | 0.000 |
| *Nephtys oligobranchia* | 0.000 | 0.000 | 0.000 | 0.000 |  | 0.000 | 0.404 | 0.732 | 0.000 |  | 0.000 | 0.000 | 0.000 | 0.000 |
| Glossiphoniidae | 0.000 | 0.000 | 0.000 | 0.000 |  | 0.038 | 0.000 | 0.000 | 0.000 |  | 0.000 | 0.000 | 0.000 | 0.000 |
| *Glossiphonia complanata* | 0.000 | 0.051 | 0.000 | 0.005 |  | 0.000 | 0.000 | 0.008 | 0.000 |  | 0.100 | 0.491 | 0.000 | 0.000 |
| *Helobdella fusca* | 0.000 | 0.000 | 0.020 | 0.000 |  | 0.000 | 0.000 | 0.000 | 0.000 |  | 0.000 | 0.000 | 0.000 | 0.000 |
| *Erpobdella octoculata* | 0.000 | 0.000 | 0.000 | 0.000 |  | 0.000 | 0.000 | 0.028 | 0.000 |  | 0.000 | 0.056 | 0.000 | 0.000 |


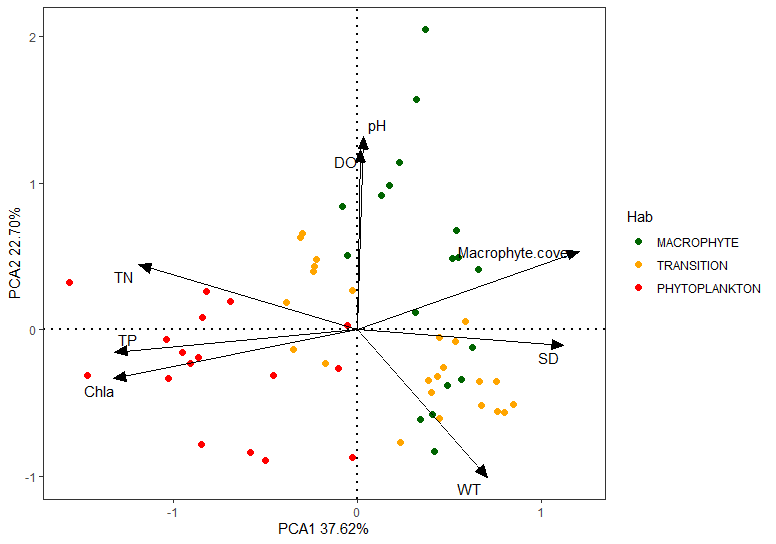


**Figure S1.** Principal Components Analysis of environmental factors in three types of lakes，*MACROPHYTE, TRANSITION* and *PHYTOPLANKTON* respectively.

**Figure S2.** Functional feeding group richness of macroinvertebrates in three types of lakes，*MACROPHYTE, TRANSITION* and *PHYTOPLANKTON* respectively.
